# NSD1: A Lysine Methyltransferase between Developmental Disorders and Cancer

**DOI:** 10.3390/life11090877

**Published:** 2021-08-25

**Authors:** Samantha Tauchmann, Juerg Schwaller

**Affiliations:** University Children’s Hospital, Department of Biomedicine, University of Basel, 4031 Basel, Switzerland; Samantha.Tauchmann@unibas.ch

**Keywords:** NSD1, H3K36, SOTOS, cancer, NUP98-NSD1, AML

## Abstract

Recurrent epigenomic alterations associated with multiple human pathologies have increased the interest in the nuclear receptor binding SET domain protein 1 (NSD1) lysine methyltransferase. Here, we review the current knowledge about the biochemistry, cellular function and role of NSD1 in human diseases. Several studies have shown that NSD1 controls gene expression by methylation of lysine 36 of histone 3 (H3K36me1/2) in a complex crosstalk with de novo DNA methylation. Inactivation in flies and mice revealed that NSD1 is essential for normal development and that it regulates multiple cell type-specific functions by interfering with transcriptional master regulators. In humans, putative loss of function NSD1 mutations characterize developmental syndromes, such as SOTOS, as well as cancer from different organs. In pediatric hematological malignancies, a recurrent chromosomal translocation forms a NUP98-NSD1 fusion with SET-dependent leukemogenic activity, which seems targetable by small molecule inhibitors. To treat or prevent diseases driven by aberrant NSD1 activity, future research will need to pinpoint the mechanistic correlation between the NSD1 gene dosage and/or mutational status with development, homeostasis, and malignant transformation.

## 1. Introduction

Gene expression is controlled by temporarily and spatially coordinated modification of chromatin. Hereby, the N-terminal tails of the histone octamers formed by H2A, H2B, H3, and H4 undergo post-translational modifications including methylation, phosphorylation, acetylation, ubiquitylation and sumoylation executed by proteins acting as “writers” of an epigenetic code [1]. Histone lysine methyltransferases (KMTs) have been characterized as critical regulators of multiple cellular processes including DNA replication, DNA damage response, cell cycle progression or cytokinesis. Genetic lesions (mutations, translocations) as well as altered gene expression functionally affecting KMTs are recurrently found in various human malignancies but also in developmental disorders [2]. An increasing number of compounds that selectively target aberrantly activated KMTs have been developed and underwent clinical trials as novel cancer therapeutics [3]. In this review, we summarize the current knowledge on the nuclear receptor binding SET domain protein 1 (NSD1, aka KMT3B), a H3 lysine 36 (H3K36) methyltransferase that has recently gained attention because of its critical role in several human pathologies, such as germline developmental syndromes and cancers.

## 2. Identification and Structure of NSD1

NSD1 was discovered in a yeast two hybrid screen for proteins associated with the ligand-binding domain (LBD) of the retinoic acid receptor alpha (RARa). NSD1 was shown to interact directly with the LBD of several nuclear receptors, including the retinoic acid (RAR), thyroid (TR), retinoid X (RXR), and estrogen (ER) receptors. These interactions are mediated by two distinct nuclear receptor interaction domains (NID) in NSD1, NID^−L^ and NID^+L^. NID^−L^ interacts with RAR and TR when a ligand is absent, whereas NID^+L^ binds RAR, TR, RXR and ER when a ligand is present, indicating that NSD1 controls repression or activation of target genes by distinct binding to nuclear receptors [4]. Similarly, a yeast-two-hybrid screen, using the LBD of the androgen receptor (AR) and the orphan receptor TR4 as baits, allowed for the detection of a human androgen receptor-associated protein of 267 Kd (ARA267) that showed the highest homology to mouse NSD1. ARA-267 (which turned out to be NSD1) was shown to be widely expressed in different tissues, with highest levels in lymph nodes. Functional studies have suggested its primary role as co-activator of AR controlled transcription [5].

**Figure 1:**
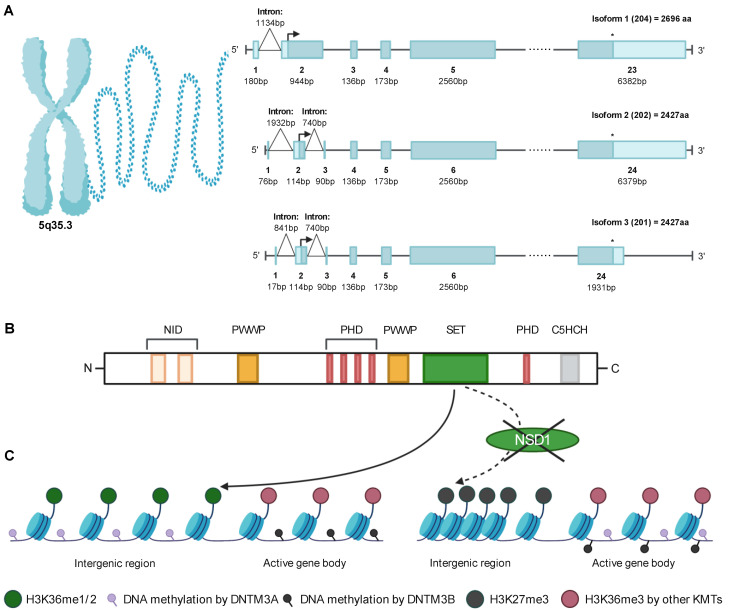
NSD1 gene and protein architecture and function. (**A**) Exon structure of the three different *NSD1* isoforms. Isoform 1 (204) contains 23 exons, whereas isoform 2 (202) and 3 (201) contain 24 exons. Open reading frame is shown by an arrow as start and asterisk at the end. (**B**) All three major NSD1 isoforms contain two nuclear receptor interacting domains (NID), two proline-tryptophan-tryptophan-proline (PWWP), five plant homodomain zinc fingers (PHD), the catalytic Su(var)3-9, enhancer-of-zeste, Trithorax (SET) and the C-terminal C5HCH (Cys-His) domain. (**C**) The NSD1 SET domain methylates H3K36me1/2 predominantly at intergenic regions allowing recruitment of DNMT3A and facilitating H3K36me3 by other KMTs allowing recruitment of DNMT3B to active gene bodies. Reduced NSD1 catalytic activity results in loss of H3K36me2 marks, which allows spreading of PRC2-mediated H3K27me3 marks at intergenic regions and redistribution of DNTM3A-mediated DNA methylation to active gene bodies.

The NSD1 gene maps to human chromosome 5q35.3, close to the telomere, with an 8088 bp open reading frame (ORF) [6]. Interrogation of ensembl.org indicates the existence of three NSD1 isoforms produced by alternative splicing, one long isoform and two shorter ones, with additional potential smaller isoforms that have been computationally mapped [7]. NSD1 isoform 1 (NSD1(204), Q96L73-1; ARA267-beta) with an ORF starting at exon 2 and ending at exon 23 has been chosen as the canonical sequence and is 2696 amino acids (aa) long, resulting in 296 kDa [6,7]. NSD1 isoform 2 (NSD1(202), Q96L73-2; ARA267-alpha) is 2427aa and 267 kDa and differs at the 5′UTR, compared to isoform 1 where 1-269aa are missing [8]. Furthermore, through an mRNA splicing event, a 740 bp long intron within exon 2 is removed, leading to an additional exon with 90 bp (exon 3), resulting in a total length of 24 exons. NSD1 isoform 3 (NSD1(201), Q96L73-3) is similar to isoform 2 with a 740 bp spliced intron; however, it differs by lacking 310-412aa, thereby resulting in a smaller intron between exon 1 and 2, with 841 bp. [7] Furthermore, exon 24 has a length of 1931 bp, which is smaller compared to isoform 2 that has a 6379 bp long exon 24. However, the ORF for both, isoform 2 and 3, starts at exon 2 and ends at exon 24, resulting in the same length and size of the protein (Figure 1A). Notably, the three isoforms (204, 202, 201) encode for proteins that contain all of the functionally characterized NSD1 domains, suggesting that variations close to the 5′ end of the ORF may be linked to regulation of gene expression.

Interrogating public databases suggests ubiquitous NSD1 expression (or its related homologs) in most tissues from various organisms. Somehow higher NSD1 mRNA levels seem to be expressed in normal brain, pancreas, male reproductive tract, and hematopoietic organs such as the bone marrow and lymphoid tissues [9]. Significant NSD1 mRNA expression in bone marrow polymorphonuclear cells, CD4, CD8 and NK cells is also supported by genevisible.com [10]. Integrated expression analysis in normal tissues and cell lines indicates abundant NSD1 protein expression in B-lymphocytes, CD8 T cells, platelets, fetal brain, retina, fetal gut, rectum, liver, adipocytes, pancreas, placenta and ovaries [11]. However, there seems to be an overall low tissue specificity for NSD1 protein expression.

The NSD1 protein contains two NIDs, two proline-tryptophan-tryptophan-proline (PWWP) domains, five plant homeodomains (PHD), an atypical (C5HCH) plant homeo-domain (PHD) finger and a catalytic domain (CD) composed of a pre-SET (AWS), Su(var)3–9, Enhancer-of-zeste, Trithorax (SET) and post-SET domain [6]. The aa sequences from both the PWWP-I and PHD-II domains are 100% identical between mouse and human NSD1, while the SET domain is 99% identical. A 97% homology between human and mouse was found for PHD-I and PHD-III. PWWP-II was 95% conserved whereas the NID^−L^ and NID^+L^ showed the least identity, with 88 and 83%, respectively [6] (Figure 1B).

NSD1 is a member of a SET-containing methyltransferase protein family, which contains two additional members, NSD2 and NSD3. Both are significantly smaller than NSD1 due to the absence of the NID^−L^ and NID^+L^ in the N-terminus. NSD2, also called Wolf-Hirschhorn Syndrome Candidate 1 (WHSC1) or Multiple Myeloma SET domain protein (MMSET) is located on the short arm of chromosome 4 (4p16.3), a locus targeted by a recurrent t(4;14)(p16;q32) translocation found in up to 20% of patients with multiple myeloma. NSD2 contains a PWWP domain, a SET domain, PHD zinc fingers and a high mobility group (HMG) box with 75% homology to NSD1 [6]. Similarly, NSD3, also called WHSC1L1, contains a PWWP, SET and PHD zinc finger domains but lacks the HMG box and is therefore only 68% identical to NSD1. NSD3 was mapped to the short arm of chromosome 8 (8p11.2), a locus involved in cancer-associated amplifications and translocations, such as t(8;11)(p11;p15) associated with myelodysplastic syndromes (MDS) and acute myeloid leukemia (AML) [12].

## 3. NSD1 Is an Epigenetic Regulator Writing and Reading Chromatin Marks

### 3.1. The SET Domain Mediates the Catalytic Activity

NSD1-3 have been functionally characterized as histone methyltransferases (HMT) due to its conserved catalytic SET domain involved in methylation of histone 3-K4, -K9, -K27, -K36, and -K79, and methylation of histone 4-K20 [13]. Members of the NSD family seem to differ from other protein lysine methyltransferases (PKMTs) as in the absence of a ligand, the SET histone binding site is closed, preventing any access to the catalytic groove [14]. In general, SET domains are approximately 130 aa long and contain binding sites for the lysine ligand and the co-factor S-adenosylmethionine (SAM), which donates methyl groups. The C-terminal post-SET domain can form a loop, thereby regulating substrate binding by forming one side of the SAM binding pocket [15]. The PWWP domains of NSD1 are critical for binding to H3K36me marks but also to DNA, whereas the PHD zinc fingers are needed for interactions with other methylated histones, such as H3K4 and H3K9 [16].

Several, mostly in vitro studies, reported other histones (H4K20) and non-histone proteins as potential NSD1 substrates. Berdasco et al. found that loss of NSD1 by 5′-CpG island DNA hypermethylation interferes with histone lysine methylation not only by decreasing the levels of H3K36me3 but also of H4K20me3 [17]. Lu et al. suggested that NSD1 acts (in tandem with the F-box and leucine-rich repeat protein 11 (FBXL11) demethylase) as a regulator of the NFκB signaling pathway indicated by reversible methylation of K218 and K221 of NFκB-p65. However, these observations were based on associations upon NSD1 overexpression or knockdown, and not validated in biochemical assays [18]. Using a biochemical approach, others were unable to validate NSD1-SET mediated methylation of H4K20 and NFκB-p65. However, they found in addition to H3K36, H1 linker histones, in particular H1.5 (K168) but also H1.2 (K168) and H1.3 (K169) as well as H4 (K44), as potential NSD1 substrates. Furthermore, they identified peptides of 50 non-histone proteins recognized by NSD1-SET. NSD1 methylation on two of those non-histone proteins, the chromatin remodeler ATRX (K1033) and the small nuclear RNA-binding protein U3 (K189), could be validated in vitro [19].

### 3.2. NSD1 Chromatin Modification and Regulation

Methylation of histone H3K36 occurs in three states mono-, di- and trimethylation and is primarily described as a hallmark of active transcription. Several KMTs were shown to be recruited by RNA polymerase II and deposit H3K36me3 over gene bodies essential for transcriptional elongation, whereas H3K36me2 is enriched at intergenic regions or promoters [20,21].

Functional studies have shown that NSD1 catalyzes mono- and dimethylation of H3K36 specifically. NSD2 leads to mono- and dimethylation of H3K36, whereas it prefers to catalyze dimethylation compared to monomethylation. Interestingly, H3K36me2 marks are not only set by NSD1-3 but also by ASH1L (ASH1 Like Histone Lysine Methyltransferase), whereas SETD2 (SET Domain Containing 2, Histone Lysine Methyltransferase) is the only enzyme able to introduce K36 methylation up to the trimethylation stage (H3K36me3) [22]. Previous studies have shown that NSD1 exhibits an autoinhibitory state that is relieved by binding to nucleosomes enabling dimethylation of histone H3 at Lys36 (H3K36) [23]. To better understand H3K36 recognition by NSD proteins, Li et al. recently solved the cryo-electron microscopy structures of mononucleosome-bound NSD2 and NSD3 [24]. They observed that binding of NSD2 and NSD3 causes DNA near the linker region to unwrap, facilitating insertion of the catalytic core between the histone octamer and the unwrapped DNA segment. Multiple DNA- and histone-specific contacts between NSD and the nucleosome precisely defined the position of the enzyme on the nucleosome.

Yuan et al. suggested that H2A mono ubiquitination (ubH2A) impairs the enzymatic activity of HMTs including NSD1, indicating another layer of complexity in NSD1 regulation [25]. Notably, ubH2A can recruit the Polycomb Repressive Complex 2 (PRC2). PRC2 regulates gene expression by methylation of lysine 27 of histone 3 (H3K27) marks through its enzymatic component EZH2 (Enhancer Of Zeste 2 Polycomb Repressive Complex 2 Subunit). The different degrees of H3K27 methylation (H3K27me1/me2/me3) have distinct genomic distributions: H3K27me1 is enriched within gene bodies of actively transcribed genes; H3K27me2 is abundant, marking 50–70% of total histone H3 and covering inter- and intragenic regions. H3K27me3 (present on 5–10% of histone H3) is strongly enriched at sites overlapping with PRC2 binding and is considered the hallmark of PRC2-mediated gene repression [26]. Streubel et al. found that genetic inactivation of *Nsd1* leads to genome-wide expansion of H3K27me3 not only at PRC2 target genes but also as de novo accumulation within broad H3K27me2 marked domains. Thus, NSD1-mediated H3K36me2 seems crucial to restrict PRC2 activity by preventing uncontrolled deposition of H3K27me3 [27].

### 3.3. Functional Interaction with DNA Methyltransferases

In addition to PRC2, epigenomic regulation by NSD1 also involves DNA methyltransferases (DNMTs), which methylate CpG dinucleotides. In total, there are five different DNMTs, of which three play a role in DNA methylation. DNMT1 is important to maintain methylation during DNA replication and acts in response to DNA damage, while DNMT3A and DNMT3B are responsible for de novo methylation [28]. DNMT3 enzymes are recruited through their PWWP domain to methylated H3K36 [29]. DNMT3B colocalizes selectively with H3K36me3 and methylates active gene bodies to enhance gene expression. DNMT3A binds more strongly to H3K36me2 than to H3K36me3 and preferentially methylates intergenic chromatin, which often co-occurs with PRC2-mediated H3K27me2 as well as NSD1-mediated-H3K36me2 [30,31]. Functional studies in ES cells revealed that ablation of NSD1 results in redistribution of DNMT3A to H3K36me gene bodies and reduced methylation of intergenic DNA [32]. Likewise, expression of a H3K36M mutant (not recognized by NSD1), resulted in an increase in H3K27me3 at intergenic regions and redistribution of PRC2 resulting in aberrant gene expression [31] (Figure 1C).

### 3.4. Regulation of Gene Expression

Depletion of NSD1 leads to both up- and down-regulation of gene expression, indicating NSD1 functions as transcriptional co-activator and co-repressor. In earlier studies, distinct stretches of the NSD1 ORF sequence were tested for their transcriptional activity by fusing them to a GAL4 DNA binding domain, which identified a region (1084–1400 aa) with a significant repressive activity in vitro. This suggested that NSD1 has a silencing domain that functions autonomously, which might act as corepressor for unliganded TR and RAR [4]. Although the mechanisms of gene repression by NSD1 are not fully understood, experimental work suggested that transcription is impaired through binding of the NSD1 C5HCH domain (adjacent to the C-terminus of PHD-V) to the C2HR zinc finger motif of ZNF496 (aka NSD1 interacting zinc finger protein 1, NIZP1) tethered on RNA polymerase II promoters [33,34]. In contrast, only expression of an N-terminal stretch of NSD1 (1–731) fused to the estrogen-receptor alpha DNA binding domain showed strong transcriptional activation in yeast but not mammalian cells [4]. More recent work demonstrated that loss of NSD1 increases H3K27ac associated with active enhancers in mESCs. NSD1 was shown to recruit the histone deacetylase 1 (HDAC1), which can deacetylate H3K27ac. Hence, inactivation of HDAC1 recapitulated increased H3K27ac similar to loss of NSD1 [35]. Overall, although these studies provided some insights into the role of NSD1 as a transcriptional co-repressor, its function as a co-activator, particularly in the context of specific nuclear receptors remains poorly understood.

## 4. Cellular Functions of NSD1

Earlier in vitro studies showed that NSD1 overexpression allowed NIH-3T3 fibroblasts to grow in reduced serum levels, whereas vector-transfected control cells did not. Overexpression of *Schizosaccharomyces pombe* SET2, which contains a SET domain but no PHD or PWWP domains, conferred reduced serum dependence, indicating that the catalytic NSD1 activity is able to modulate serum dependence [36].

### 4.1. Modeling NSD1 Activity in the Fly

To better understand the function of NSD1 in vivo, gain- and loss-of-function studies in various organisms have been performed. Ubiquitous NSD (the fly NSD1 homolog) overexpression in *Drosophila melanogaster* caused developmental delay and reduced body size at the larval stage, resulting in pupal lethality. Targeted overexpression in various tissues led to significant alterations that rescued RNAi-based NSD knockdown. NSD overexpression enhanced the transcription of pro-apoptotic genes and led to caspase activation. Notably, NSD-overexpression associated wing atrophy was reduced by a loss-of-function mutation in Jun N-terminal (JNK) kinase [37]. NSD1 overexpression in *Drosophila* imaginal discs induced organ atrophy. Interestingly, ectopic expression of the DNA replication-related element-binding factor (DREF) resulted in increased NSD expression [38]. DREF proteins are central regulators of cell proliferation; however, whether the human homolog ZBED1 (zinc finger BED-type-containing 1) regulates NSD1 expression remains unknown. Pan-glial, but not pan-neuronal NSD overexpression induced apoptosis in *Drosophila* larval brain cells. However, pan-glial NSD overexpression also induced caspase-3 cleavage in neuronal cells. Among the various glial cell types, NSD overexpression in only astrocytic glia induced apoptosis and abnormal learning defects in the larval stage. These observations in *Drosophila* suggested that aberrant NSD expression may result in neurodevelopmental disorders through functional interference with astrocytes [39]. In contrast, NSD deletion by CRISPR/Cas9-mediated knock-out resulted in an increase in the body size of *Drosophila* larvae. Although the NSD mutant flies survived to adulthood, their fecundity was dramatically decreased. NSD lacking flies also showed neurological dysfunctions, such as lower memory performance and motor defects, and a diminished extracellular signal-regulated kinase activity [40]. Collectively, these functional studies in the fly suggested that NSD is a central regulator of proliferation and, cell and/or body size.

### 4.2. Modeling NSD1 Activity in the Mouse

To gain insight into the biological functions of NSD1 in mammals, Losson and colleagues have generated mice carrying a floxed *Nsd1* exon 5 containing the nuclear factor interaction domain. Ubiquitous inactivation (*Actin-iCre;Nsd1^f/f^*) embryos displayed a high incidence of apoptosis and failed to complete gastrulation, indicating that NSD1 is essential for early post-implantation development [41]. More recent work, using the same *Nsd1^f/f^* allele, showed that conditional targeted ablation in primordial germ cells (*Tnap-iCre;Nsd1^f/f^*) resulted in male sterility associated with absence of mature spermatozoa and loss of testicular germ cells in adult testis and epididymis. A similar effect was seen when DNMT3A was conditionally ablated in germ cells. Male mutant mice presented with impaired spermatogenesis due to loss of methylation at two out of three paternally imprinted loci in spermatogonia [42]. Molecular studies confirmed previous findings that NSD1 safeguards a subset of genes against H3K27me3-associated transcriptional silencing. In contrast, H3K36me2 in oocytes is predominantly dependent on the SETD2 HMT coinciding with H3K36me3. Hence, in contrast to males, *Nsd1^−/−^* females are fertile. These studies showed that NSD1 plays a critical role in the maturation of mouse gametes by regulating distinct profiles of H3K36 methylation [43]. A third study using the floxed *Nsd1* mouse allele generated by Losson et al. inactivated the gene in the hematopoietic system. Unexpectedly homozygous ablation during late fetal liver hematopoiesis (*Vav-iCre;Nsd1^f/f^*) resulted in a fully penetrant hematological malignancy phenocopying many aspects of human acute erythroleukemia. Functional studies revealed that lack of *Nsd1* impairs terminal differentiation of erythroblasts, which could be rescued by expression of wildtype, but not a catalytically inactive SET-domain NSD1*^N1918Q^* mutant. Interestingly, NSD1, but not the inactive mutant, significantly increased the occupancy of the erythroid transcriptional master regulator GATA1 at target genes and their expression. These studies identified NSD1 as a novel regulator of GATA1-controlled erythroid differentiation [44]. Very recently, Zou and coworkers used the same floxed murine *Nsd1* allele for targeted activation of the gene in mesenchymal progenitor cells (*Prx1-iCre*;*Nsd1^fl/fl^*). Ablation of *Nsd1* in mesenchymal progenitors resulted in impaired cartilage development, skeletal growth defects, and impaired fracture healing. Chondrogenic differentiation was impaired, which was associated with reduced H3K36me2 marks and lower expression of critical mediators including the SRY-box transcription factor 9 (SOX9). Interestingly, in chondrocytes NSD1 seems to bind the promoter and to control expression of the hypoxia-inducible factor 1alpha (HIF1alpha*)*, a well-known regulator of SOX9 [45]. Importantly, *Sox9* overexpression rescued the chondrogenic differentiation effects of *Nsd1^−/−^* cells. Collectively, these data suggest that NSD1 controls chondrogenic differentiation by direct (H3K36me2) and indirect (HIF1A) regulation of SOX9 [46]

Piper and colleagues used a CRISPR/Cas9 strategy to inactivate exon 3 of *Nsd1*. Although they did not find any major morphologic defects in *Nsd1^+/−^* brains, the animals exhibited deficits in social behavior without significant learning or memory deficits. *Nsd1^−/−^* E9.5 embryos had a smaller prosencephalon compared to heterozygous and wildtype animals, with abnormal morphology and aberrant formation of the luminal cavity of the brain [47]. Taken together, NSD1 inactivation studies in *Drosophila* and mice showed that NSD1 is essential for normal development and that it regulates a wide variety of cellular functions, of which many seem to be cell type-specific, most likely by controlling the activity of distinct transcriptional master regulators.

## 5. Role of NSD1 in Human Diseases

### 5.1. Aberrant NSD1 Activity Is a Hallmark of Developmental Syndromes

Germline lesions (including missense, truncating and splice-site mutations and submicroscopic deletions) potentially resulting in loss-of-function of the NSD1 protein have been linked to a developmental syndrome called SOTOS [48]. SOTOS is a childhood overgrowth syndrome characterized by a distinctive facial appearance, physical overgrowth with height and head circumference >97th percentile, advanced bone age and learning disabilities [49]. Interestingly, microduplications of 5q35.2–q35.3 encompassing the *NSD1* gene locus have been reported in rare patients with a clinically reversed SOTOS syndrome. These individuals are characterized by short stature, microcephaly, learning disability or mild to moderate intellectual disability, and distinctive facial features. These observations suggest that the NSD1 gene dosage determines the phenotype of these developmental syndromes [50].

Analysis of a cohort of >700 individuals with overgrowth and intellectual disability revealed a putative causal mutation in less than 15 genes in almost half of the individuals [51]. Notably, epigenetic regulation was a prominent biological process not only represented by NSD1 but also by five additional genes including PRC2 complex proteins (EZH2, EED), H1.5 linker histone (HIST1H1E), the de novo DNMT3A methyltransferase, and the chromatin remodeler CHD8. Other patients had mutations in genes controlling cellular growth (PTEN, AKT3, PIK3CA, MTOR, PPP2R5D). The PI3K/AKT pathway is a central regulator of growth by increased cell metabolism, survival, and turnover, as well as protein synthesis. As deregulated cellular growth is a hallmark of cancer, and certain human overgrowth syndromes are associated with increased cancer risk, it is not unexpected that the majority of the mutated genes in overgrowth syndromes including NSD1, EZH2, DNMT3A, PTEN, CHD8, HIST1H1E, MTOR, PIK3CA are also frequently altered in human cancers [51]. Interestingly, overgrowth-related PIK3CA mutations were shown to exhibit a striking allele dose-dependent stemness phenotype in human pluripotent stem cells (PSC) [52,53]. Whether NSD1 mutations affect PSC stemness remains unknown.

Analysis of genome-wide DNA methylation of SOTOS syndrome patients revealed a highly specific signature able to differentiate patients with pathogenic NSD1 mutations from controls, benign NSD1 variants and clinically overlapping syndromes. This NSD1^+/−^ DNA methylation signature encompasses genes that function in cellular morphogenesis and neuronal differentiation reflecting cardinal features of SOTOS syndrome [54]. SOTOS-related DNA methylation signatures were used to model epigenetic clocks that predict biological age. The so-called Horvath epigenetic clock model revealed that NSD1 loss-of-function mutations substantially accelerate epigenetic aging [55].

### 5.2. Aberrant NSD1 in Human Cancers

The first evidence linking NSD1 genetic aberrations to cancer came from cloning of a cytogenetically silent t(5;11)(q35;115) chromosomal translocation associated with pediatric de novo MDS or aggressive AML that leads to fusion of the N-terminal domains of the nucleopore 98 (NUP98) protein to the C-terminal part (including the SET) of NSD1 [56]. Importantly, in most patients, additional genetic lesions are found in NUP98-NSD1^+^ AML cells of which activating FLT3-ITD mutations are by far the most prevalent, present in about 80% of the cases [57]. Reconstitution of lethally irradiated mice with bone marrow retrovirally overexpressing the NUP98-NSD1 fusion (in presence or absence of a functionally cooperating FLT3-ITD mutation) was reported to induce an AML-like disease in mice [58,59]. Functional studies suggested that the NUP98-NSD1 fusion binds genomic elements adjacent to the *HoxA7* and *HoxA9* loci and maintains histone H3K36 methylation and histone acetylation, preventing transcriptional repression of the *HoxA* gene cluster during differentiation. Structure functional analysis indicated that the phenylalanine-glycine (FG) repeats of the NUP98 moiety as well as the NSD1-SET domain are necessary for its transforming activity [58]. Targeted sequencing of a large number of genes associated with hematologic malignancies revealed rare and potentially deleterious *NSD1* mutations in AML patients suggesting that not only gain but also loss of NSD1 can contribute to transformation of hematopoietic cells [60].

Analysis of cancer-associated aberrant CpG promoter methylation revealed epigenetic silencing of NSD1 in human brain tumor cell lines associated with reduced H3K36 methylation [17]. While NSD1 overexpression impaired colony growth in semi-solid medium and proliferation of cancer cells, RNAi-mediated knock-down increased proliferation, suggesting a role of a tumor suppressor [17]. Frequent NSD1 epigenetic silencing was also found in human clear cell renal cell carcinoma (ccRCC). Notably, tumors harboring NSD1 promoter methylation were of higher grade and stage, and NSD1 promoter methylation correlated with somatic mutations in the SETD2 H3K36me3 HMT. Interestingly, ccRCC with epigenetic NSD1 silencing displayed a specific genome-wide methylome signature consistent with the NSD1 mutation methylome signature observed in SOTOS syndrome [61]. Comprehensive genomic characterization of human head and neck squamous cell carcinomas (HNSCC) identified inactivating NSD1 mutations and focal homozygous deletions in up to 10% of the patients [62]. Further studies revealed recurrent mutations including a K36M oncomutation in multiple H3 histone genes. Interestingly, direct in vitro inhibition of NSD2 and SETD2 by H3K36M has been described, whereas inhibition of NSD1 was only found in steady-state kinetic analysis using inhibitory H3 (27–43) peptide containing K36M [63,64]. Notably, along with previously described NSD1 mutations, they corresponded to a specific DNA methylation cluster. In addition, the K36M substitution and NSD1 defects converged on altering methylation of H3K36, subsequently blocking cellular differentiation and promoting oncogenesis [62]. Extensive genetic analysis of HNSCCs revealed that, similar to what has been experimentally observed in ES cells, loss of function NSD1 mutations are responsible for reduced intergenic H3K36me2 marks, followed by loss of DNA methylation and gain of H3K27me3 in the affected genomic regions. Those regions seem enriched in cis-regulatory elements, and subsequent loss of H3K27ac correlated with reduced expression of putative target genes [65]. In addition to HNSCC, H3.3 K36M mutations are recurrently found in several rare human cancers including chondroblastomas and poorly differentiated sarcomas. Comparison of the epigenomic and transcriptomic landscape of mesenchymal cells experimentally depleted of H3K36me2 indicated recapitulation of H3K36M’s effect on H3K27me3 redistribution and gene expression [66]. Notably, transgenic mice overexpressing H3.3K36M in the hematopoietic system developed a lethal phenotype characterized by blocked erythroid differentiation that was very similar to that reported upon conditional *Nsd1* inactivation again supporting the converting consequences on epigenomic regulation [44,67].

A similar hypomethylated tumor subtype enriched for inactivating NSD1 mutations and deletions was also found in lung squamous cell carcinoma (LUSC). NSD1-altered HNSCC and LUSC correlated at the DNA methylation and gene expression levels, featuring ectopic expression of developmental transcription factors and genes that are also hypomethylated in SOTOS syndrome. Reduced expression of NSD1 was also reported to be part of an epigenetic gene signature able to distinguish non-malignant tumor from tissue of prostate cancer. Surprisingly, metastatic lesions appeared to express significantly higher NSD1 levels than primary tumors [68]. Highly prevalent NSD1 mutations were also found in testicular germ cell tumors, and low NSD1 expression was associated with resistance to cisplatin [69]. However, the functional significance of NSD1 alterations in human urogenital cancers remains to be investigated.

Comprehensive genomic analysis of 21 tumor types originating from >6000 samples revealed that the degrees of overall methylation in CpG island and demethylation in intergenic regions, defined as the ‘backbone’, are highly variable between different tumors [70]. Interestingly, NSD1 mutations showed the most significant association with backbone DNA demethylation not only in HNSCC but also in other cancers. In fact, bi-allelic NSD1 aberrations by mutation or gene copy loss showed the highest backbone demethylation [70]. A computational search for cancer predisposition genes based on the Knudson’s two-hit hypothesis using genome data of ~10,000 tumors identified genes including *NSD1* that may contribute to cancer through a combination of rare germline variants and somatic loss-of-heterozygosity (LOH). Interestingly, rare germline variants in such genes may contribute substantially to cancer risk, particularly of ovarian carcinomas, but also other cancers [71].

Researchers also explored the correlation between allele frequency of somatic variants and total gene expression of the affected gene using matched tumor and normal RNA and DNA sequencing data from almost 400 individuals across 10 cancer types. They defined higher allele frequency of somatic variants in cancer-implicated genes. This study revealed that somatic alleles bearing premature terminating variants (PTVs) in cancer implicated genes seemed to be less degraded via nonsense-mediated mRNA decay, possibly favoring truncated proteins. Notably, NSD1 appeared as a gene with more than five somatic variants and PTVs with high allele frequency [72].

**Figure 2:**
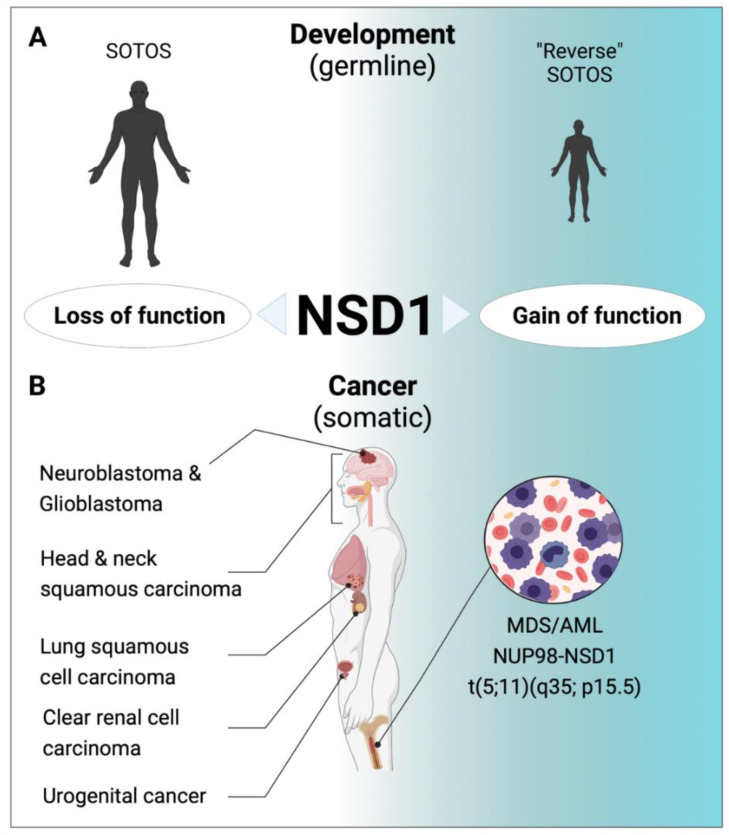
Role of NSD1 in human diseases. (**A**) Inactivating mutations in NSD1 are the molecular hallmark of SOTOS syndrome, a developmental disorder characterized by a distinctive facial appearance, physical overgrowth advanced bone age and learning disabilities [48,49]. “Reverse” SOTOS Syndrome is characterized by a short stature, microcephaly, and learning disability, and is associated with microdeletions of 5q35 carrying NSD1 [50]. (**B**) Putative loss of function mutations of NSD1 are among the most prevalent lesions in human head and neck and lung squamous cell carcinomas, neuroblastomas and glioblastomas [17,61,62,65,66,68]. NSD1 gene silencing was found in human clear cell renal cell carcinoma, and urogenital cancers [69,71]. In pediatric myeloid malignancies (de novo MDS and AML) the chromosomal translocation t(5;11)(q35;115) results in expression of a NUP98-NSD1 fusion gene with SET-dependent leukemogenic activity [56,57,58,59].

Collectively, increased NSD1-SET activity drives a particular hematological cancer, whereas loss-of function mutation or impaired expression characterize a wide variety of mostly solid human cancers (Figure 2).

## 6. Therapeutic Interference with NSD1

Several strategies have been explored to selectively interfere with NSD1 activity. Earlier work characterized a small molecule methyltransferase inhibitor (BIX-01294) able to modulate H3K9 methylation. BIX.01294 was characterized as a G9a inhibitor by binding to the histone-tail groove in the SET domain [73]. Notably, BIX-01294 was also found to differentially inhibit NSD1, NDS2 and NSD3 in vitro based on the structural conserved catalytic SET domain but the molecule clearly lacks any NSD1 specificity [74].

NSD1 contains several PHD zinc fingers, whereby the PHD-V C5HCH domain serves as a binding site for protein–protein interactions. This region is particularly interesting as it has been shown to be involved in dysregulated Hox gene activation in AML and occurrence of point mutations in SOTOS Syndrome [16]. PHD-V C5HCH recruits a transcriptional repressor, resulting in a direct finger–finger interaction with the C2HR domain of Nizp1 [34]. The consequences of this binding are not clear; therefore, interfering with this interaction would be interesting to elucidate the biological and pathological relevance. Targeting PHD fingers has been considered to pharmacologically interfere with protein function; however, the affinity of compounds to specifically target a particular region is not advanced enough to be implemented in vivo. Berardi et al. designed a computational and experimental pipeline to investigate the druggability by using a 3D model of the PHD-V C5HCH domain of NSD1 with the C2HR domain of Nizp1 [34]. Applying a structure-base in silico screening following NMR validation, they found three structurally related molecules that were able to bind to the PHD-V C5HCH domain of NSD1: type II topoisomerase inhibitor mitoxantrone, chloroquine and quinacrine. Even if these compounds are interesting to target the NSD1/Nizp1 interaction, the consequences of derepressing transcription and selective inhibition are not clear and more functional studies have to be performed before this can be translated into the clinic.

Using a luminescence screening platform that quantifies S-adenosyl homocysteine (which is produced during methyl transfer from S-adenosylmethionine used by NSD1 and other HMTs), researchers identified suramin and other scaffolds as potential inhibitors of the enzymatic NSD1 HMT activity [75]. A computational strategy incorporating ligand contact information into classical alignment-based comparisons applied to SET containing proteins revealed additional scaffolds that inhibited NSD1 activity [76].

More recently, Grembecka, Cierpicki and colleagues employed a fragment-based screening strategy to identify and optimize first-in-class irreversible small-molecule inhibitors of the NSD1 SET domain [77]. Structural analysis revealed that NSD1 in complex with covalently bound ligands results in a conformational change in the autoinhibitory loop of the SET domain and formation of a channel-like pocket suitable for targeting with small molecules. Importantly, their lead-compound (“BT5”) demonstrated on-target activity in NUP98-NSD1 immortalized cells associated with reduction of H3K36me2 and downregulation of critical target genes, such as the *HOXA* gene cluster and *MEIS1*. Notably, BT5 also impaired the clonogenic growth of primary NUP98-NSD1^+^ AML cells but not leukemic cells carrying an KMT2A-MLLT1 fusion or normal human CD34^+^ hematopoietic stem and progenitor cells. The discovery of this compound provides a platform for the development of potent and selective NSD1-SET inhibitors [77].

## 7. Outlook

It is well established that NSD1 regulates gene expression programs through H3K36 methylation in a complex crosstalk between activating and repressive histone marks, as well as DNA methylation. In addition, NSD1 is a target of recurrent germline or somatically acquired loss and gain-of-function alterations associated with developmental syndromes (e.g., SOTOS) and various human cancers. However, many open questions remain; in particular, it is currently poorly understood how a putative loss of function mutation or reduced expression results in the observed developmental and cancer phenotype.

Molecular characterization of SOTOS patient-derived DNA confirmed the connection between NSD1 loss-of-function mutations and aberrant DNA CpG methylation [54]. However, it seems unclear whether SOTOS is based on simple NSD1 haploinsufficiency, or whether particular mutants eventually have dominant-negative activity, functionally impairing the protein expressed from the unmutated allele. Some studies have suggested an increased risk for SOTOS patients to develop cancer, raising the question about the role of NSD1 mutations in this context. As the cancer risk in SOTOS patients is small, one wonders whether further reduction of the NSD1 gene dosage (by, e.g., epigenetically silencing of the wildtype allele) could be involved [78]. A better characterization of the gene dosage and protein activity relationship in developmental syndromes is necessary to explore whether the presence of NSD1 mutations can serve more than as a diagnostic marker but eventually also provide some translational opportunities [79].

As outlined before, predicted loss-of-function mutations or epigenetic silencing of NSD1 have been described in a variety of human cancers. Investigating HNSSC or lung squamous cell carcinomas revealed that mutations did not abrogate NSD1 expression in most samples. Notably, interrogation of the cancer cell line encyclopedia (CCLE) indicates that only a very small number (5/1457) of human cancer cell lines completely lost NSD1 expression at the mRNA level [80,81]. Currently, it remains unclear how a single NSD1 point mutation will contribute to malignant transformation. Is further reduction of the NSD1 gene dosage, e.g., by loss of heterozygosity (LOH), necessary to significantly enhance malignant transformation? Notably, heterozygous *Nsd1^+/−^* mice do not develop any pathologies and express normal *Nsd1* mRNA and protein levels [44]. In addition, we observed that shRNA-mediated knockdown experiments only significantly affected growth of various human and mouse cells after reduction of NSD1 mRNA levels over 50% (unpublished data). Hence, a systematic analysis of the functional *NSD1* gene dose in malignant transformation is necessary. It also remains unresolved whether genetic alterations are early or late events in cancer development.

In addition, the critical downstream effectors of the tumor suppressive activity of NSD1 remain unknown. Although recent molecular analysis of human HNSCC cancer cell lines with and without NSD1 mutations (generated by CRISPR/Cas9 genome editing) revealed aberrant regulation of genes related to oxidative phosphorylation, MYC, mTORC1 or RAS signaling and other pathways, the impact on the cell biology has not been addressed and no particular transformation effector genes have been validated [65]. In addition, further studies are necessary to show whether the disease phenotypes with aberrant functional NSD1 dose are purely the consequence of its chromatin regulatory role or whether yet to be identified non-chromatin NSD1 substrate proteins are critically involved [3].

Notably, one of the most significantly down-regulated pathways in HNSSC cells, carrying engineered NSD1 mutations, was interferon alpha/gamma signaling [65]. Earlier studies identified human HNSCC and lung squamous cell carcinoma enriched for NSD1 inactivating mutations and deletions that displayed an immune-cold phenotype characterized by low degree of infiltration by tumor-associated leukocytes (macrophages, CD8^+^ T cells) as well as low expression of immune checkpoint ligands and receptors (PD1, PDL1, PDCD1LG2) [82]. Interestingly, tumors formed by lung cancer cell lines with shRNA-mediated reduced NSD1 expression in immunodeficient mice contained also less tumor-infiltrating T cells and were associated with reduced expression of various cytokines and chemokines [82]. Another study proposed that a chemokine expression signature allows classification of HNSCC into high and low CD8^+^ T cell-infiltrated tumor phenotypes (TCIP-H vs. TCIP-L) associated with different clinical outcome. Notably about 20% of TCIP-L tumors carried loss of function NSD1 mutations [83]. These observations suggest that human cancers may escape the immune system through acquisition of NSD1 mutations. Further work is necessary to dissect the cellular and molecular circuits of cell-autonomous from non-autonomous consequences of aberrant NSD1 activity in human diseases. Interestingly, in vitro functional studies performed with human brain and breast cancer cells lines found a potential link of reduced expression or mutations of NSD1 to drug resistance; however, its general significance for cancer therapy remains to be validated [84,85].

When fused to the N-terminus of NUP98, the NSD1-SET gains transforming activities in hematopoietic cells, resulting in myelodysplasia and AML, and the presence of a NUP98-NSD1 (and other NUP98-fusions) is often associated with primary resistance to chemotherapy [86,87]. Functional studies suggested that transformation by these fusions involves the NUP98-GFLG repeats recruiting a large WDR82-SET1A/B-COMPASS protein complex to promote H3K4 trimethylation and favor active transcription [88]. The fusions may also directly interact with KMT2A (aka MLL1) to reach critical target gene loci such as the *HOX-A* gene cluster regulated by the fusion partner like NSD1 that favors transcription by H3K36 methylation [58,89]. These findings strongly suggest that targeted inactivation of the NSD1-SET domain shows anti-leukemic activity in NUP98-NSD1^+^ hematological malignancies.

Although selective NSD1-SET inhibitors are highly relevant for aggressive NUP98-NSD1^+^ pediatric AML, one has to take into consideration that loss-of-function mutations of NSD1 are much more prevalent in human cancers. Will such NSD1-SET inhibitors also block NSD1’s role as a tumor suppressor? Significantly reduced NSD1 activity may functionally affect transcription factors controlling maturation of hematopoietic cells (and eventually also cells from other tissues). In the best-case scenario, some reduction of the NSD1-SET might be sufficient to induce differentiation of NUP98-NSD1-transformed myeloid cells, whereas significant side effects (as observed in gene targeted mice) may only develop upon complete inactivation over a longer time period that will most likely never be reached by such compounds.

The future NSD1 research agenda should aim to (i) mechanistically determine the gene dosage–phenotype correlation in germline syndromes with aberrant NSD1 activity, (ii) identify the cellular and molecular mechanisms of malignant transformation by altered NSD1 activity (mutations, epigenetic silencing), and (iii) optimize and validate small molecule NSD1-SET inhibitors for therapy of pediatric AML, driven by the NUP98-NSD1 fusion gene, and research for strategies to selectively interfere in situations when reduced NSD1 activity is the driving force.
